# Pressure-stabilized superconductive yttrium hydrides

**DOI:** 10.1038/srep09948

**Published:** 2015-05-05

**Authors:** Yinwei Li, Jian Hao, Hanyu Liu, John S. Tse, Yanchao Wang, Yanming Ma

**Affiliations:** 1 School of Physics and Electronic Engineering, Jiangsu Normal University, Xuzhou 221116, China; 2 State Key Laboratory of Superhard Materials, Jilin University, Changchun 130012, China; 3 Department of Physics and Engineering Physics, University of Saskatchewan, Saskatoon, S7N 5E2, Canada

## Abstract

The search for high-temperature superconductors has been focused on compounds containing a large fraction of hydrogen, such as SiH_4_(H_2_)_2_, CaH_6_ and KH_6_. Through a systematic investigation of yttrium hydrides at different hydrogen contents using an structure prediction method based on the particle swarm optimization algorithm, we have predicted two new yttrium hydrides (YH_4_ andYH_6_), which are stable above 110 GPa. Three types of hydrogen species with increased H contents were found, monatomic H in YH_3_, monatomic H+molecular “H_2_” in YH_4_ and hexagonal “H_6_” unit in YH_6_. Interestingly, H atoms in YH_6_ form sodalite-like cage sublattice with centered Y atom. Electron-phonon calculations revealed the superconductive potential of YH_4_ and YH_6_ with estimated transition temperatures (*T*_c_) of 84–95 K and 251–264 K at 120 GPa, respectively. These values are higher than the predicted maximal *T*_c_ of 40 K in YH_3_.

The study of hydrogen-rich compounds at hig pressure is mostly motivated by their potential high-tempeature superconducivities at high pressures. In 2004, Ashcroft^1^ suggested that hydrogen-rich compounds can become metallic and superconducting at lower pressures than hydrogen, preumably because of “chemical compression”. Since then, theoretical studies have revealed a large number of superconducting hydrides (SiH_4_^2^,^3^,^4^, SnH_4_^5^,^6^, GeH_4_^7^, ScH_3_^8^, YH_3_^9^, GaH_3_^10^, H_2_S^11^ *et al.*) at high pressures with predicted *T*_c_ ranging from 17 to 86 K. Remarkably, our previously prediction of high *T*_c_ (80 K at 160 GPa) in H_2_S^11^ has been proven recently from experiments^12^. Recently, a new type of hydrogen solvated molecular complex SiH_4_(H_2_)_2_ has been synthesized^13^,^14^ and was predicted to have a *T*_c_ of ~100 K at 250 GPa^15^, which is much higher than the 17 K observed in SiH_4_^2^.The experimental and theoretical studies have led to further investigations on hydrides with large hydrogen fraction that may provide a pathway to better superconductors. Using first-principle structure predictions, Zurek *et al.*^16^ first predicted three new lithium hydrides (LiH_2_, LiH_6_ and LiH_8_) above 130 GPa, which are stabilized by charge transfer from Li to H. Subsequent studies have revealed a number of hydrides with large hydrogen fractions at high pressures, such as Na-H^17^, K-H^18^,^19^, Rb-H^20^, Cs-H^21^, Ca-H^22^, GeH_4_-H_2_^23^ and H_2_S-H_2_^24^. Remarkably, some hydrides were predicted to possess high *T*_c_, e.g., ~82 K in LiH_6_ (300 GPa)^25^, ~70 K in KH_6_ (166 GPa)^18^ and strikingly ~235 K in CaH_6_ (150 GPa)^22^.

Among the different hydrides, yttrium hydrides (YH_*n*_) are of special interest because each Y atom has three valence electrons and, in principle, could be shared with three H atoms. Experimentally, that during the continuous absorption of H atoms, a reversible transition of YH_*n*_ between the reflecting YH_2_ and optically transparent YH_3_ was observed. This interesting phenomenon offers a great potential for practical application as a “switchable mirror”. Raman^26^ and infrared^27^ studies found that the semiconducting YH_3_ transforms to a metallic fcc structure above 10 GPa. Singnificantly, fcc-YH_3_ was predicted to be a superconductor with *T*_c_ of 40 K at 17.7 GPa, the lowest reported pressure for hydrides to date^9^. The prediction, however, has not been confirmed by experiment.

At high pressure, it is expected that the valence electronic state of Y atom will change and therefore provides a possibility of bonding with more H atoms. Here, we focus on the formation of Y hydrides with larger H concentration at high pressures of YH_*n*_ (*n* = 2, 3, 4, 5, 6, 8). Two new thermodynamically stable hydrides with stoichiometries of YH_4_ and YH_6_ were found above 110 GPa. Electron-phonon calculations show both YH_4_ and YH_6_ are superconductive with relatively high *T*_c_.

## Results

**Figure 1:**
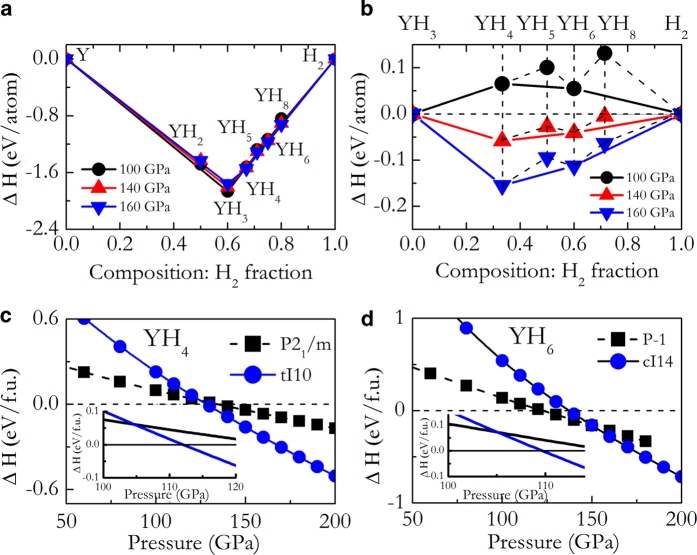
Thermodynamical stability of new yttrium hydrides at high pressure. Formation enthalpies (Δ*H*) of various Y-H stoichiometries with respect to (Y + solid H_2_, a) and (YH_3_ + solid H_2_, b) at 100, 140 and 160 GPa. Δ*H* in (**a**) and (**b**) were calculated with equations of Δ*H* = *H*_YH*n*_ – (*H*_Y_ + *nH*_H2_/2) and Δ*H* = *H*_YH*n*_ – (*H*_YH3_ + (*n*-3)*H*_H2_/2, respectively. (**c**) and (**d**) present the static enthalpy curves of various structures of YH_4_ and YH_6_ relative to (YH_3_ + solid H_2_) as functions of pressure without (main figures) and with (insets) zero-point energy corrections, respectively.

The enthalpies of the candidate structures of YH_*n*_ found in structure predictions relative to the products of dissociation into Y + solid H_2_ and YH_3_ + solid H_2_ at selected pressures are summarized in Fig. 1 (a,b), respectively. Fig. 1 (a) shows all the stoichiometries considered here possess negative formation enthalpies with respect to Y + solid H_2_. Among those, YH_3_ has the lowest energy. Two thermodynamically stable polymorphs, YH_4_ and YH_6_, are to be thermodynamically more stable than the decomposition into YH_3_ + sold H_2_ were found at 140 and 160 GPa (Fig. 1b). Although YH_2_, YH_5_ and YH_8_ have negative formation enthalpies with respect to Y + solid H_2_, they are expected to decompose at all pressure. For example, YH_2_ decomposes into YH_3_ + Y since the enthalpy is above the tie-line connecting YH_3_ and Y. Similarly, YH_5_ and YH_8_ decompose into YH_4_ + YH_6_ and YH_6_ + H_2_, respectively (Fig. 1b). Therefore,YH_2_, YH_5_ and YH_8_ are excluded in the discussions hereafter.

**Figure 2:**
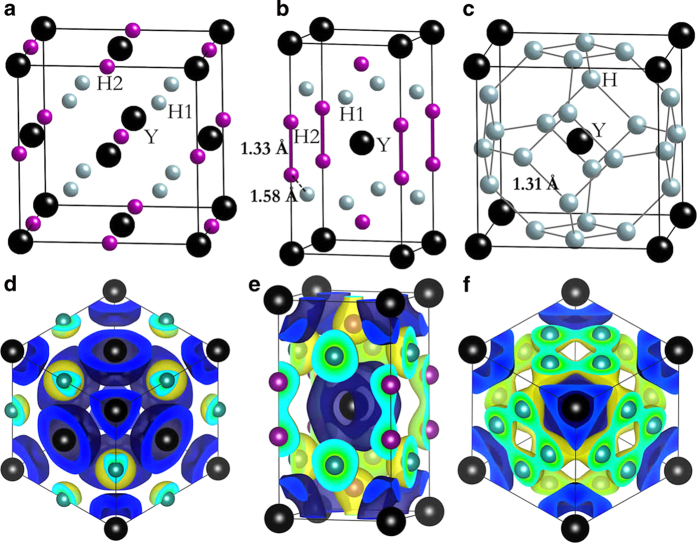
High pressure crystal structures of yttrium hydrides. Crystal structures of fcc-YH_3_ (**a**) tI10-YH_4_ (**b**) and cI14-YH_6_ (**c**). The lattice parameters of tI10-YH_4_ at 120 GPa are *a* = 2.87 Å and *c* = 5.33 Å with Y atom sitting at 2*a* (0, 0, 0) and two nonequivalent H1 and H2 atoms at 4*d* (0.5, 0, 0.75) and 4*e* (0, 0, 0.63), respectively. For cI14-YH_6_ at 120 GPa, *a* = 3.7 Å, Y and H atoms occupy 2*a* (0, 0, 0) and 12*d* (0, 0.5, 0.25) positions, respectively. (**d**–**f**) are three-dimensional charge density difference with isosurface value of 0.01 *e*/Bohr^3^ of fcc-YH_3_, tI10-YH_4_ and cI14-YH_6_, respectively. Blue and yellow colors represent losing and gaining electrons, respectively.

So far, YH_3_ is the only experimentally known yttrium hydrides at high pressure. The structure search readily reproduced the observed fcc structure^26^,^27^ at 100 and 150 GPa. As can be seen from Fig. 2 (a), there exist only one type of Y atom occupying the fcc site and two nonequivalent, H1 and H2, atoms at the octahedral and tetrahedral sites. The H-H separation is of 1.9 Å at 120 GPa, clearly indicates no interaction between the H atoms (Fig. 2d). Therefore, the H atoms in fcc-YH_3_ are monoatomic. fcc-YH_3_ was previously predicted to be stable in a large pressure region of 20 GPa^28^ to 197 GPa^29^ and undergo a superconductor – metal – superconductor transition under pressure^9^.

Figure 1 (c,d) show the formation enthalpies of YH_4_ and YH_6_ with respect to YH_3_ + solid H_2_ as functions of pressure. The formation enthalpy of YH_4_ becomes negative relative to YH_3_ + solid H_2_ near 128 GPa (Fig. 1c). It is important to include the quantum nuclear zero-point energies (ZPE) when considering the energetics of systems containing light atoms. We therefore calculated the ZPEs of YH_4_, YH_3_ and H_2_ phases within the quasi-harmonic approximation. When ZPE corrections are included, the predicted pressure for the onset of stability of YH_4_ is lowered to 112 GPa (inset in Fig. 1c). The stable YH_4_ has a tetragonal structure (space group *I*4/*mmm*, denoted tI10 hereafter, Fig. 2b) with two formula units per unit cell. We found that the tI10-YH_4_ has the same structure type with tI10-CaH_4_^22^. The tI10 structure at 120 GPa consists of body-centered arranged Y atoms and two nonequivalent H1 and H2 atoms with H1-H2 and H2-H2 distances of 1.58 and 1.33 Å, respectively. Valence electrons localization was found between the two neighbouring H2 atoms while absent between H1 and H2 atoms (Fig. 2e). This indicates the presence of both molecular “H_2_” and monoatomic H in tI10-YH_4_.

YH_6_ becomes thermodynamically more stable than YH_3_ + solid H_2_ above 122 GPa (Fig. 1d). The onset pressure of the stability of YH_6_ is reduced to 110 GPa when considering the ZPE effect (inset in Fig. 1d). In the thermodynamically stable pressure region, YH_6_ adopts a cubic structure with space group *Im*-3*m* (2 f.u./unit cell, denoted cI14 hereafter, Fig. 2c). The “H_6_” hexagons are forming a corner-shared sodalite-like cage with a Y atom at the center, the same sodalite structure found in CaH_6_ above 150 GPa^22^. In this case, the H-H distance of 1.31 Å at 120 GP is longer than in CaH_6_. Despite the longer distance, covalent interaction between H atoms is clearly visible from the localized valence electrons between the H atoms (Fig. 2f).

## Discussion

We found three types of H species in YH_n_ compounds, monatomic H in YH_3_, monatomic H+molecular “H_2_” in YH_4_ and hexagonal “H_6_” in YH_6_. Since molecular H_2_ has a filled covalent *σ* bond, the additional electrons donated from Y will occupy the antibonding σ* bond, resulting in a stretched or even dissociated H-H bond. For example, the formation of YH_3_ can be described by the reaction 2Y + 3H_2_ → 2YH_3_. Assuming all 6 valence electrons (3 from each Y atom) were transferred to the H_2_, then, each H_2_ would accommodate two additional electrons into the σ* orbital and, thus, breaking the H_2_ molecule into monatomic H. Integration of the electron density shows that each H1 (H2) atom in fcc-YH_3_ have accommodated an additional 0.54 (0.47) electrons. A similar description can be used for the formation of YH_4_. In this the reaction is Y + 2H_2 _→ YH_4_. There are three electrons available to two H_2_ molecules. Therefore, one H_2_ bond is completely broken into two monoatomic H and the remaining electron occupied the σ* one the second H_2_, thereby weakening the bond and resulted in a longer H-H distance of 1.33 Å. The additional charge of the monatomic H1 in tI10-YH_4_ is calculated to be 0.42 electrons. Only 0.29 electrons were added to H2 in tI10-YH_4_. Finally, the formation of YH_6_ can be described as Y + 3H_2 _→ YH_6_. In this case only, one electron is added to each H_2_ and the H-H bond is elongated to 1.31 Å, in close agreement with the “molecular” H_2_ in YH_4_. In cI14-YH_6_, each H atom has accepted 0.25 electrons, which is not enough to dissociate the H_2_ molecule.

**Figure 3:**
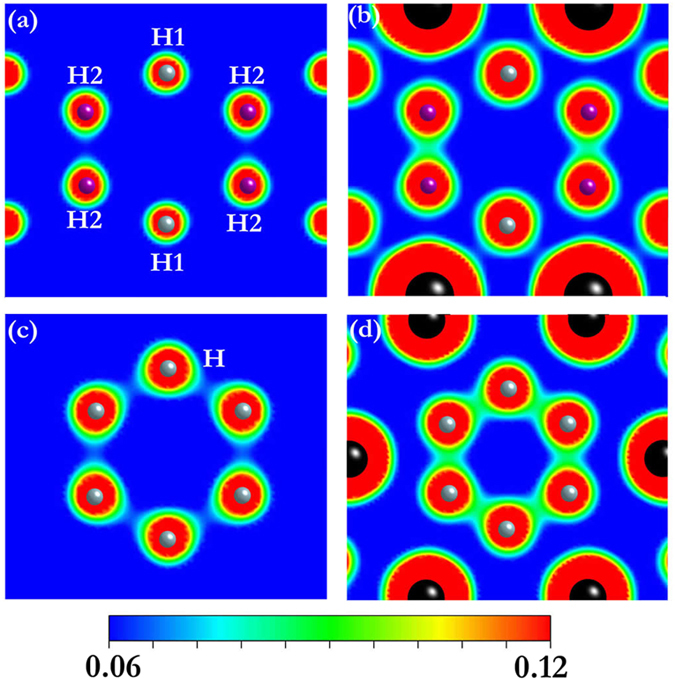
Valence charge densities. The valence charge densities of H sublattice in tI10-YH_4_ (**a**) tI10-YH_4_ (**b**) H sublattice in cI14-YH_6_ (**c**) and cI14-YH_6_ (**d**) within the plane containing “H_2_” or “H_6_” hexagons at 120 GPa.

A previous study^22^ has shown that 4*s*-3*d* charge transfer turns Ca from *s*-dominant into *s*-*d* dominant at high pressure, similar to the electronic configuration of Y atom. Therefore, the presence of same structure types in YH_4_ (YH_6_) and CaH_4_ (CaH_6_) is not accidental. However, the H-H distance of “H_2_” molecule in YH_4_ (1.33 Å) is much longer than that (0.81 Å) in CaH_4_ at 120 GPa as Y transfer one more valence electron to H_2_ than Ca resulting in a longer H-H distance in YH_4_ (YH_6_). The empirical consideration is support from quantitative calculations of the difference of the electron density of tI10-YH_4_ to that of a hypothetical structure consisting only H sublattice. It is clearly shown in Fig. 3(a,b), that there is no electron density (covalent bond) between the two H2 atoms in the pure H structure. However, when the Y atoms were present, localized electrons are found between two H2 atoms. Therefore, the charge transfer from Y to H2 is responsible to the formation of molecular “H_2_” in YH_4_. Similarly, the foramtion of “H_6_” hexagons in YH_6_ results from the accommodation by H of excess electrons from Y atom (Fig. 3c,d). For comparison, a survey of molecular “H_2_” in most hydrogen-rich compounds only show a slightly elongated H-H bond length than pure solid H_2_. Examples are 0.87 Å in GeH_4_^7^, 0.79 Å in SnH_4_^6^, 0.84 Å in SiH_2_(H_2_)_2_^15^, 0.76 Å in LiH_2_^16^ and 0.8 Å in NaH_9_^17^.

**Figure 4:**
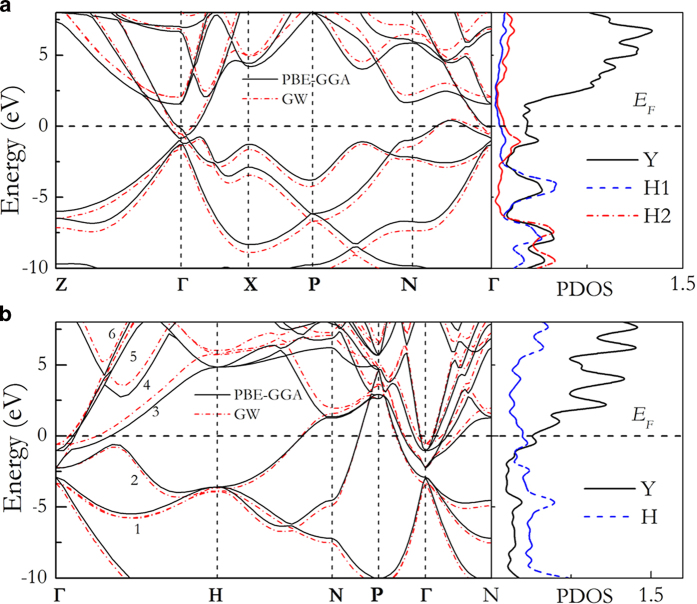
Electronic band structures and projected density of states (PDOS). Electronic band structures and PDOS (in units of eV^−1^ per f.u.) of tI10-YH_4_ (**a**) and cI14-YH_6_ (**b**) at 120 GPa. Dashed red lines in left pannels are the *GW*-corrected band structures. The horizontal dashed lines represent Fermi level (*E*_F_). The numbers (1–6) in (**b**) label the six bands accrossing the Fermi energy.

The electronic properties, lattice dynamical and electron-phonon coupling parameter (EPC) of tI10-YH_4_ and cI14-YH_6_ have been calculated. Both tI10-YH_4_ and cI14-YH_6_ are found to be metals from the band structures presented in Fig. 4. We found three features common to tI10-YH_4_ and cI14-YH_6_: (i) the large densiy of states at the Fermi level (*N*_F_), 0.44 eV^−1^ per f.u. in tI10-YH_4_ and 0.6 eV^−1^ per f.u. in cI14-YH_6_ at 120 GPa; (ii) the concurrence of falt and steep electronic bands near the *N*_F_, providing a possibility of the pairing of eletrons at the *N*_F_^30^; (iii) strong Y-H hybridization derived from the significant overlap of Y- and H-DOS. Note that in a preivous study^9^ it was demonstrated that the Y-H hybridization is responsible for the superconductivity in YH_3_.

**Figure 5:**
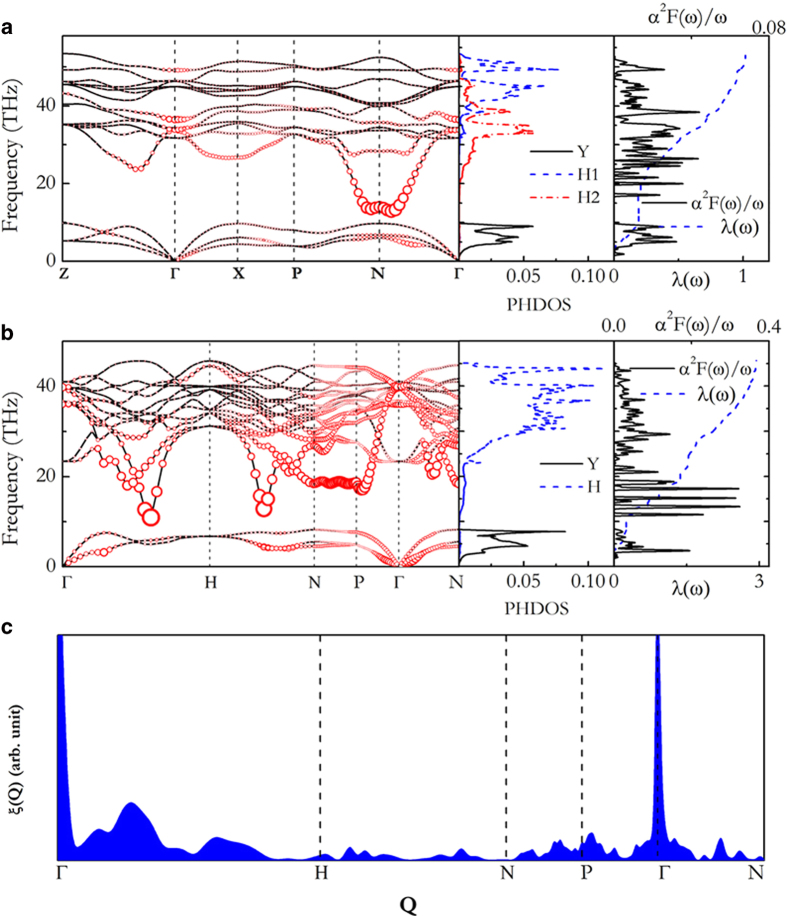
Phonon properties and Eliashberg spectral function. Phonon dispersions, projected phonon density of states (PHDOS), Eliashberg spectral function *α*^2^F(ω)/ω and EPC integration of λ(ω) of tI10-YH_4_ (**a**) and cI14-YH_6_ (**b**) at 120 GPa. Red circles in the two left pannels indicate the phononline width with a propotional to the strength. (**c**) **The nesting function of cI14-YH**_**6**_ along several high-symmetry lines of Q calculated at 120 GPa. The present calculation employs 3094 *k* points and 126 *Q* points, which result in the evaluaion of energy ε_*k* + *Q*_ at 390000 points.

**Figure 6:**
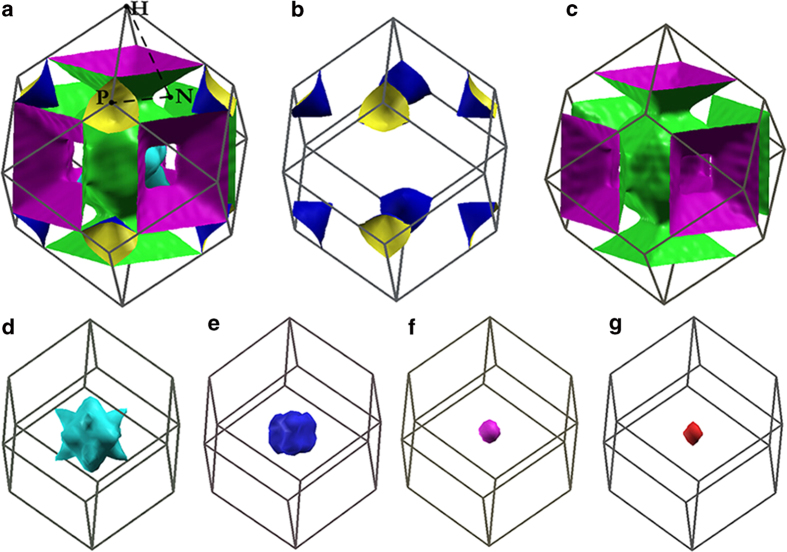
Fermi surface of cI14-YH_6_. The Fermi surface of cI14-YH_6_ calculated at 120 GPa. (**a**) The 3D view of the Fermi surface including all cutting bands. (**b**)-(**g**) The Fermi surface of each band acrossing the Fermi energy. The Fermi surface is sampled with a 20 × 20 × 20 *k* mesh.

Figure 5 shows the calculated phonon dispersions, phonon density of states (PHDOS), Eliashberg spectral function (*α*^2^F(ω)/ω)and EPC integrated (λ(ω))for tI10-YH_4_ and cI14-YH_6_ at 120 GPa. The absence of any imaginary phonon modes proves the dynamical stabilities of both compounds. As expected, both phonon spectra are separeted into two frequency regions, with the low frequencies (<10 THz) dominated by the vibrations of Y atom while the high end of the spectra by H atoms. In tI10-YH_4_, the resulting EPC parameter λ is 1.01 at 120 GPa, which is comparable to the maximum value (~1.4^9^) predicted for fcc-YH_3_. Note that the low-frequency vibrations contribute to 18% of the total λ while the ramaining 82% comes from H vibrations. Circles with radius proportional to the EPC were also plotted in Fig. 6 to illustrate the contributions associated with different phonon modes. One can observe that nearly all phonon modes contribute to the overall λ, reflecting a three-dimensional nature of the structure.

Surprisingly, according to the calculation, the EPC parameter λ of cI14-YH_6_ reaches 2.93 at 120 GPa, even larger than that (2.69 at 150 GPa^22^) in cI14-CaH_6_. However, the Eliashberg phonon spectral functions of cI14-CaH_6_ and cI14-YH_6_ are quite different. The EPC in cI14-CaH_6_ was derived primarily from the two phonon modes (*T*_2g_ and *E*_g_) at the zone center Γ point. However, we observed an overall contribution of different modes to λ along N-P-Γ-N directions. Moreover, 90% of the total λ is contributed by H vibrations. The superconductivity in YH_6_ is associated with the Kohn anomalies observed in the phonon dispersion of the phonon branch Γ-H and H-N. The calculation of the nesting function (Fig. 5c) confirms this expectation and clearly show strong nesting along Γ-H and H-N directions. Compare to the other 5 bands crossing the Fermi level, the Fermi surface of strongly nested band (Fig. 6c) shows a complex “vase”-like topology with strong nesting along Γ-H.

*T*_c_ was estimated from the spectral function (*α*^2^F(μ)) by numerically solving the Eliashberg equations^31^ with typical choice of Coulomb pseudopotential μ* = 0.1−0.13. The Coulomb repulsion is taken into account in terms of the μ* scaled to a cutoff frequency^32^. At 120 GPa, the calculated *T*_c_ is 84–95 K for tI10-YH_4_, much higher than the maximal 40 K predicted for fcc-YH_3_^9^. Note that tI10-YH_4_ has a much larger logarithmic average frequency of 1119 K than fcc-YH_3_ (350 K^9^) due to the presence of molecular “H_2_”, which helps to enhance the superconductivity. For cI14-YH_6_, *T*_c_ value of 251–264 K was estimated. This value is comparable to the predicted *T*_c_ (220–235 K at 150 GPa) in CaH_6_^22^. Although, in principle, there is no upper limit to the *T*_c_ value within the Midgal-Eliashberg theory, remarks on the very high *T*_c_ value of cI14-YH_6_ must be view with caution. The EPC calculations were based on the harmonic approximation and without the consideration of electron correction effects. A previous study^38^ had shown that anharmonicity of atomic motion may reduce or even suppress the superconductivity of AlH_3_ due to the renormalization of the lower vibration modes by anharmonicity^33^. However, this suggestion is contrary to the observation that anharmonic vibraions will significantly enhance *T*_c_ in case of disordered compounds^34^. Another important, but often neglected, situation is that the Fermi level topology may be altered in improved electronic band structure including corrections to self interaction and electron correlation effects. In AH_3_, the parallel bands favouring nesting disappeared in the GW calculated band structure^35^. Here, *GW* band struture calculations were performed for tI10-YH_4_ and cI14-YH_6_. No significant change in the band structures, particularly for the bands near or crossing the Fermi level, was found in both case (Fig. 4). Therefore, the discussion presented above will still be valid and we expect YH_4_ and YH_6_ are good superconductors.

## Methods

Structure predictions for YH_*n*_ were performed using the particle swarm optimization technique implemented in the CALYPSO code^36^,^37^. In recent studies, it was shown that the approach was successful on the prediction of high pressure structures on both elemental and binary compounds, such as N^38^, Ca-H^22^, H_2_S^11^ and BeH_2_^39^. In this work, systematic structure search were performed on six stoichiometries (YH_2_, YH_3_, YH_4_, YH_5_, YH_6_ and YH_8_) at 100 and 150 GPa. Model cells up to 4 formula units (f.u.) for each stoichiometry were used. The structure search was considered converged when~1000 successive structures were generated after a lowest energy structure was found.

*ab initio* structure relaxations were performed using density functional theory within the Perdew-Burke-Ernzerhof (PBE) generalized gradient approximation (GGA) as implemented in the Vienna *ab initio* simulation package (VASP)^40^. The band structures were calculated with both PBE-GGA and *GW* methods^41^. The *GW* interpolated band structures were computed using WANNIER90^42^. The all-electron projector augmented wave (PAW)^43^ method was adopted with 1*s* and 4*s*^2^4*p*^6^4*d*^1^5*s*^2^ treated as valence electrons for H and Y, respectively. An energy cutoff of 700 eV and a Monkhorst-Pack Brillouin zone sampling grid with a resolution of 0.5 Å^−1^ were used in the structure searches. Selected low energy structures were then re-optimized with a denser grid better than 0.2 Å^−1^ and a higher energy cutoff of 1000 eV. Phonon dispersion and electron-phonon coupling (EPC) calculations were performed with density functional perturbation theory using the Quantum-ESPRESSO package^44^. Norm-conserving pseudopotentials for Y and H were considered with a kinetic energy cutoff of 140 Ry. 8 × 8 × 8 (59 *q*-points) and 10 × 10 × 10 (47 *q*-points) *q*-meshes in the first Brillouin zones were used in the EPC calculations for YH_4_ and YH_6_, respectively. Monkhorst-Pack grids of 32 × 32 × 32 and 40 × 40 × 40 were used to ensure *k*-points sampling convergence with Gaussians of width 0.03 Ry for YH_4_ and YH_6_, respectively, in order to approximate the zero-width limit in the calculations of the EPC parameter, λ.

## Conclusion

In conclusion, structure predictions have demonstrated that yttrium atom can react with more than three hydrogens under pressure. Two high-hydride phases, YH_4_ and YH_6_, were predicted to be thermodynamically stable relative to YH_3_ and H_2_ above 110 GPa. At the stable pressure ranges, YH_4_ has a bct strucure containing both moniatomic H and molecular “H_2_” while YH_6_ adopted a bcc structure with a H sodalite-like cage. Electron-phonon coupling calculations show that both YH_4_ and YH_6_ are supercodncutive with *T*_c_ higher than YH_3_. The results presented here support the suggestion that compressing the mixture of elements (compounds) and hydrogen is a way to search high-temperature superconductors. In addition, in principle, YH_4_ and YH_6_ can be synthesized by compressing the mixture of YH_3_ and H_2_ above 110 GPa.

## Author Contributions

Y. L. and Y. W. conceived the idea. Y.L., J. H. and H. L. performed the calculations. Y L. and J. T. and Y. M. wrote the manuscript with contribution from all.

## Additional Information

**How to cite this article**: Li, Y.* et al.* Pressure-stabilized superconductive yttrium hydrides. *Sci. Rep.* 5, 9948; doi: 10.1038/srep09948 (2015).
